# The effects of anodal-tDCS on corticospinal excitability enhancement and its after-effects: conventional vs. unihemispheric concurrent dual-site stimulation

**DOI:** 10.3389/fnhum.2015.00533

**Published:** 2015-09-30

**Authors:** Bita Vaseghi, Maryam Zoghi, Shapour Jaberzadeh

**Affiliations:** ^1^Faculty of Medicine, Department of Physiotherapy, School of Primary Health Care, Nursing and Health Sciences, Monash UniversityMelbourne, Australia; ^2^Department of Medicine, Royal Melbourne Hospital, The University of MelbourneParkville, Australia

**Keywords:** unihemispheric concurrent dual-site anodal transcranial direct current stimulation, primary motor cortex, corticospinal excitability, pain neuromatrix, neuroplasticity, long-lasting effect

## Abstract

Previous researchers have approved the ability of anodal transcranial direct current stimulation (a-tDCS) of the primary motor cortex (M1) to enhance corticospinal excitability (CSE). The primary aim of the current study was to investigate the effect of concurrent stimulation of M1 and a functionally connected cortical site of M1 on CSE modulation. This new technique is called unihemispheric concurrent dual-site a-tDCS (a-tDCS_UHCDS_). The secondary aim was to investigate the mechanisms underlying the efficacy of this new approach in healthy individuals. In a randomized crossover study, 12 healthy right-handed volunteers received a-tDCS under five conditions: a-tDCS of M1, a-tDCS_UHCDS_ of M1-dorsolateral prefrontal cortex (DLPFC), a-tDCS_UHCDS_ of M1-primary sensory cortex (S1), a-tDCS_UHCDS_ of M1-primary visual cortex (V1), and sham a-tDCS_UHCDS_. Peak-to-peak amplitude of transcranial magnetic stimulation (TMS) induced MEPs, short-interval intracortical inhibition (SICI) and intracortical facilitation (ICF) were assessed before and four times after each condition. A-tDCS_UHCDS_ conditions induced larger MEPs than conventional a-tDCS. The level of M1 CSE was significantly higher following a-tDCS_UHCDS_ of M1-DLPFC than other a-tDCS_UHCDS_ conditions (*p* < 0.001), and lasted for over 24 h. The paired-pulse TMS results after a-tDCS of M1-DLPFC showed significant facilitatory increase and inhibitory change. A-tDCS_UHCDS_ of M1-DLPFC increases M1 CSE twofold that of conventional a-tDCS. A-tDCS_UHCDS_ of M1-DLPFC enhances the activity of glutamergic mechanisms for at least 24 h. Such long-lasting M1 CSE enhancement induced by a-tDCS_UHCDS_ of M1-DLPFC could be a valuable finding in clinical scenarios such as learning, motor performance, or pain management. The present study has been registered on the Australian New Zealand Clinical Trial at http://www.anzctr.org.au/ with registry number of ACTRN12614000817640.

## Introduction

Anodal transcranial direct current stimulation (a-tDCS) of the primary motor cortex (M1) is a well-known technique (Nitsche and Paulus, 2000, 2011; Stagg and Nitsche, 2011) for modulating the resting membrane potentials of neurons, resulting in alteration of the endogenous excitability of brain neural circuits and networks (Medeiros et al., 2012). Recent fMRI studies showed that a-tDCS increases corticospinal excitability (CSE) of both local stimulated and distant areas, probably through interconnections between them (Meyerson et al., 1993; Lang et al., 2005). Literature indicates that tDCS induces CSE enhancement in M1, which could be used as a priming or stand-alone technique in therapeutic scenarios including improvement of motor function (Goodwill et al., 2013; Williams et al., 2013; Dutta et al., 2014; Filmer et al., 2014; Ludemann-Podubecka et al., 2014), motor learning (Kuo et al., 2008; Stagg and Nitsche, 2011; Zimerman et al., 2012; Karok and Witney, 2013; Vollmann et al., 2013; Meinzer et al., 2014; Parasuraman and Mckinley, 2014), and pain management (Bolognini et al., 2013; Bae et al., 2014; Foerster et al., 2015; Hagenacker et al., 2014; Moloney and Witney, 2014; Vaseghi et al., 2014; Wang et al., 2014; Zmigrod, 2014).

The focus of a large number of tDCS studies is to identify the optimal a-tDCS parameters for induction of larger CSE with longer lasting effect compared to conventional tDCS approach. Despite some promising results from previous studies, which investigated the effects of current densities/intensities (Furubayashi et al., 2008; Moliadze et al., 2014; Murray et al., 2014), electrode size (Nitsche et al., 2007; Kronberg and Bikson, 2012; Bastani and Jaberzadeh, 2013), the number of within-session repetitions of a-tDCS (Bastani and Jaberzadeh, 2012), and the duration of tDCS application (Nitsche and Paulus, 2001; Furubayashi et al., 2008), additional exploratory studies are needed to refine the existing parameter and to introduce a novel tDCS approach. One important tDCS parameter is the electrode montage. Conventional tDCS montage involves the application of the anode over a presumed target (e.g., M1 for CSE enhancement) and the cathode over an indifferent cortical site, i.e., contralateral supraorbital area. In addition to the conventional electrode montage, some clinical researchers introduced a single channel bi-hemispheric montage (Vines et al., 2008). In this montage, the reference electrode (cathode) is located over the contralateral M1. The aim is to reduce inhibition from the contralateral M1 and to induce larger CSE under the anode (Vines et al., 2008; Kidgell et al., 2013b; Park et al., 2014; Koyama et al., 2015). However, due to the reduction of M1 CSE under the cathode, the applicability of this approach for cortical (Mordillo-Mateos et al., 2012; O’Shea et al., 2014) or behavioral (O’Shea et al., 2014) modifications has not been widely accepted yet.

Apart from M1 stimulation for induction of CSE changes, research interest has shifted toward stimulation of cortical sites, which are functionally connected to M1, including dorsolateral prefrontal cortex (DLPFC), primary sensory cortex (S1), or premotor cortex. This new approach is backed by the result of some fMRI studies, which showed that the excitability modulation induced by a-tDCS is not limited to the stimulated sites; functionally connected areas are also affected (Lang et al., 2005; Kwon et al., 2008; Keeser et al., 2011). For instance, a-tDCS of the premotor cortex (Boros et al., 2008), S1 (Kirimoto et al., 2011), or DLPFC (Vaseghi et al., 2015b) increases M1 CSE. In addition, literature approved he involvement of both S1 and DLPFC in two networks involved in planning, execution, and control of movements (Kandel, 2000; Miller, 2000; Saper et al., 2000; Miller and Cohen, 2001; Hasan et al., 2013; Borich et al., 2015) and pain management (Apkarian et al., 2005; Iannetti and Mouraux, 2010). The results of these studies provide evidence for functional relationship among these cortical sites.

Therefore, unilateral concurrent stimulation of M1 and its functionally connected cortical sites would be a possible alternative electrode montage for induction of larger M1 CSE with longer lasting effects compared to conventional a-tDCS electrode montage. This new technique was called unihemispheric concurrent dual-site a-tDCS (a-tDCS_UHCDS_). The rationale behind the superiority of this new approach is that a-tDCS_UHCDS_ intensifies the mutual communications between M1 and its functionally connected sites (Luft et al., 2014). Therefore, this pilot study aimed to compare the potential effects of a-tDCS_UHCDS_ of M1-S1 and M1-DLPFC with the conventional M1 a-tDCS on CSE enhancement and its lasting effects.

We hypothesised that a-tDCS_UHCDS_ induces larger and longer-lasting CSE than conventional electrode montage of M1 a-tDCS. Due to the novelty of the proposed technique, we also aimed to investigate the possible mechanisms behind the a-tDCS_UHCDS_-induced CSE changes. Drawing on the basic mechanisms behind the efficacy of conventional a-tDCS (Liebetanz et al., 2002; Hummel et al., 2005, 2010; Nitsche et al., 2005; Paulus et al., 2008; Medeiros et al., 2012; Kidgell et al., 2013a), we hypothesised that a-tDCS_UHCDS_ of M1 and the other functionally connected site of M1 decreases short-interval intracortical inhibition (SICI), and increases ICF. Similar to conventional a-tDCS, a-tDCS_UHCDS_ involves the application of low-amplitude current via surface electrodes, which is expected to be tolerable for the participants. However, as tDCS_UHCDS_ is a new neuromodulatory approach, we also aimed to assess its possible side effects.

## Material and Methods

### Study Design

We implemented a sham-controlled crossover study to determine the effect of a-tDCS_UHCDS_ on M1 CSE in healthy individuals. All experimental procedures were approved by the Monash University Human Research Ethics Committee and conformed to the Declaration of Rohrich (2007). The current study is registered as a clinical trial on the Australian New Zealand Clinical Trial (registry number: ACTRN12614000817640)^1^.

### Participants

Twelve healthy (nine women and three men, all Monash University students) with mean age of 25 ± 1.31 (age range 19–36 years) participated in all experimental sessions. The sample size was calculated (with power of 80%) based on the data generated from the first six participants. All were right-handers as determined by the Edinburgh Handedness Inventory (10-item version, mean laterality quotient = 89 ± 9.3; Oldfield, 1971). None of the participants reported contraindications to transcranial magnetic stimulation (TMS) or tDCS, current use of any medications, or history of neurological or psychiatric disease. The health condition of participants was assessed before written informed consent was sought and provided. All volunteers were blinded to the purpose of the experiments.

### Assessment of CSE of M1

CSE of M1 was measured by the peak-to-peak amplitude of TMS-induced motor-evoked potentials (MEPs) of the right first dorsal interossei (FDI) muscle. Single- and paired-pulse magnetic stimuli were delivered by a MagPro R30 (MagOption) stimulator (MagVenture, Denmark) with an angulated figure-of-eight coil (max. initial dB/dt 28 KT/s near the coil surface). The coil was placed over left M1, contralateral to the target muscles, with a posterior-anterior orientation, and set at angle of 45° to the midline. The area of stimulation with largest MEPs was defined as the hotspot and marked on the scalp to be used throughout the tests to ensure consistency of the coil placement. Resting motor threshold (RMT) was defined as the minimal stimulator output needed to elicit five MEPs in a series of 10 with minimum amplitude of 50–100 μV in the relaxed FDI muscle (Rossini et al., 1994; Hallett, 1996; Wassermann et al., 2008). Single-pulse MEPs were recorded with the TMS intensity adjusted to elicit ~1 mV peak-to-peak amplitude at baseline. Stimulation intensity was kept constant for the post-intervention assessments.

### Assessment of Intracortical Inhibition and Facilitation

In order to evaluate the function of intracortical inhibition and facilitation circuits in M1, paired-pulse TMS was used to measure SICI and intracortical facilitation (ICF; Kujirai et al., 1993). In this method, a subthreshold TMS stimulus is followed by a suprathreshold TMS pulse with an inter-stimulus interval (ISI) of 1–5 ms or 8–15 ms to measure SICI or ICF respectively (Kujirai et al., 1993). In the present study, conditioning stimulus intensity was applied as 80% of RMT (0.8 × RMT), followed by a suprathreshold test stimulus (Di Pino et al., 2014). The test stimulus intensity was adjusted to achieve a baseline MEP of around 1 mV (Zoghi et al., 2003; Kothari et al., 2014). The ISI was set at 3 ms to measure SICI and 10 ms to measure ICF (Di Pino et al., 2014; Opie and Semmler, 2014). Five blocks of ISI were designed to deliver both single- and paired-pulse TMS randomly. Each block contained 20 single-pulse and 40 paired-pulse TMS (20 ISI of 3 and 20 ISI of 10 ms). One of five blocks was randomly selected in each time point of measurement to minimize the bias induced by the order of stimuli. Blocks of MEPs in which the muscle was not relaxed were excluded from the analysis. In order to avoid any profound effect of inter-pulse interval on MEP size, a ten-second interval was applied between stimulations (Vaseghi et al., 2015a).

### tDCS Characteristics

**Figure 1:**
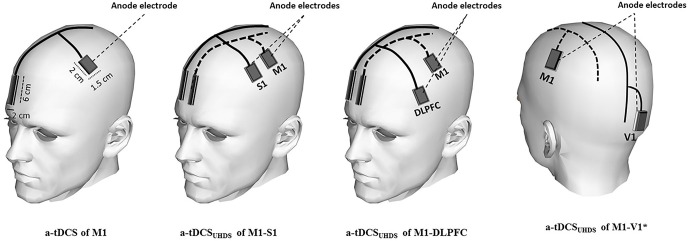
**Schematic illustration of electrode montage in conventional a-tDCS and a-tDCS_UHCDS_; the active electrodes were positioned over M1, dorsolateral prefrontal cortex (DLPFC), S1, and V1**. The reference electrodes were placed over the contralateral supraorbital area in all conditions. In the sham condition, the electrodes were placed in the same positions as for M1-S1 or M1-DLPFC stimulation. *The reference electrodes are not shown in a-tDCS_UHCDS_ of M1-V1.

Participants received tDCS under each of five different conditions in random order: a-tDCS of M1, a-tDCS_UHCDS_ of M1-S1, a-tDCS_UHCDS_ of M1-DLPFC, a-tDCS_UHCDS_ of M1-V1, and sham a-tDCS_UHCDS_. Direct current was applied through active saline-soaked surface sponge electrodes (1.5 × 2 cm) over target areas including M1, S1 and DLPFC, and reference electrodes (2 × 6 cm) over the contralateral supraorbital area (Bikson et al., 2010; Figure 1). The small size of active electrode produces a highly focused DC current over the target areas, which enabled us to stimulate M1 and S1 with two separated anode electrodes separately. Based on the result of some computational modeling studies, the effects of tDCS can be more focalized by smaller electrodes (Nitsche et al., 2007; Bikson et al., 2010). In addition, recent experimental investigations on human brain illustrated that utilizing smaller active electrodes over M1 resulted in larger CSE (Nitsche et al., 2007; Bastani and Jaberzadeh, 2013; Vaseghi et al., 2015b).

The tDCS stimulators were set to deliver 0.3 mA direct current for 20 min, with 10 s of linear fade in and fade out. Current intensity of 0.3 mA allowed us to considerably decrease the size of electrodes (Uy and Ridding, 2003) while keeping the current density in a safe range (0.1 mA/cm^2^) with limited side effect (Poreisz et al., 2007; Brunoni et al., 2011). The superiority of lower intensities in induction of larger CSE has been shown by some tDCS studies (Nitsche and Paulus, 2001; Brunoni et al., 2011; Parazzini et al., 2013; Pellicciari et al., 2013).

Two channels of a tDCS device were used for stimulation of the target areas in a-tDCS_UHCDS_ conditions. Current intensity of 0.3 mA and density of 0.1 mA/cm^2^ were identical in each active electrode during all experimental conditions.

Using a similar electrode montage as conventional tDCS protocols, the narrow shaped cathodal reference electrodes were placed over contralateral supraorbital area over subgenual cortex (Figure 1). To reduce the neuromodulatory effects of these electrodes, the size of them were kept four times larger than the active electrodes. This arrangement considerably reduces the density under these electrodes.

The anode was placed over the left M1 for the right FDI muscle as identified by TMS. For stimulation of S1, the anode was identified based on the international 10–20 system and the anode was placed over C′3 (2 cm posterior to C3). For a-tDCS of DLPFC and the primary visual cortex (V1), the anode was placed over F3 and Oz respectively (Figure 1). The reference electrode (cathode) was conventionally placed over the contralateral supraorbital area with the assumption of no or negligible neuromodulatory effects on the subgenual cortex. As V1 is not directly connected to M1, a-tDCS_UHCDS_ of M1–V1 was a control condition to assess whether the changes following a-tDCS_UHCDS_ of M1-DLPFC or M1–S1 are due to stimulation of the brain with twice the current density of conventional a-tDCS or concurrent stimulation of M1 and a functionally connected site to the M1. In the sham condition, the electrodes were placed in the same positions as for M1-S1 or M1-DLPFC stimulation randomly, but the stimulator was turned off after 30 s of stimulation. All pre and post evaluations were identical to those in other conditions.

### Experimental Procedure

Using a cross-over study design, each participant was randomly assigned to receive all active and sham conditions. We allocated a code for each participant and experimental condition. Using a random number table, the sequence of experimental conditions was assigned for 12 participants and placed in opaque envelops to ensure the concealment of the allocation. Then, one envelop was allocated to each participants’ code in a random order. The volunteers were comfortably seated in a fully adjustable treatment chair (MagVenture, Denmark) with head and arm rests. First, the hotspot of M1 FDI was identified by single-pulse TMS and marked. Then the stimulus intensity was adjusted to elicit single-pulse MEPs with peak-to-peak amplitudes of an average of 1 mV. After determination of RMT, 80% of RMT was calculated as the subthreshold test stimulus. Twenty single-pulse MEPs and 40 MEPs induced by paired-pulse TMS, including 20 MEPs with ISI of 3 and 20 MEPs with ISI of 10 ms, were recorded. The single- and paired-pulse TMS with ISI of 3 and 10 ms were applied in a random order.

**Figure 2:**
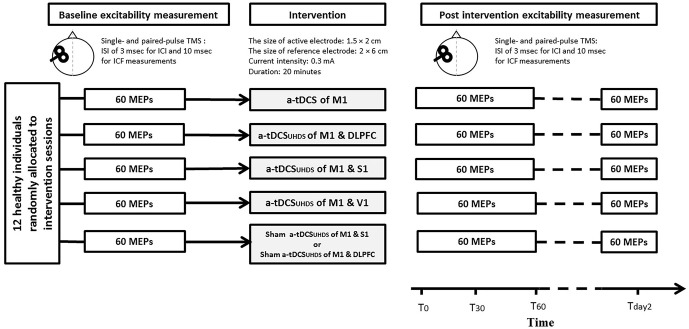
**Experimental design for the comparison of conventional a-tDCS and a-tDCS_UHCDS_; in each time point of measurement, 20 single pulse transcranial magnetic stimulation (TMS), 20 paired pulse TMS with inter-stimulus interval (ISI)s of 3 ms, and 20 paired-pulse TMS with ISIs of 10 ms were delivered to measure corticospinal excitability (CSE), short interval intracortical inhibition (SICI) and ICF respectively**.

Based on the participant code and the assigned sequence, tDCS was applied in each experimental session. The experimental sessions were separated by at least 7 days to avoid interference or carry-over effects of tDCS, and completed at the same time of the day (late mornings or early afternoon) to avoid diurnal variation. The duration of tDCS application was 20 min in all experiments. All the outcome measures were measured before (T_pre_), immediately after (T_0_), 30 min (T_30_) and 60 (T_60_) minutes after each intervention. TMS measurements were conducted 24 h after the end of the intervention (T_day2_; Figure 2). To control the effect of female hormonal fluctuation on the size of MEPs, the experimental sessions were carried out between the 7th and 21st day of women’s menstrual cycles. Participants were blinded to the condition of tDCS (sham or active).

### Measurement of Side Effects

To record side or adverse effects of stimulation, all participants were asked to complete a questionnaire during all experimental conditions. The questionnaire contained rating scales for the presence and severity of side effects such as itching, tingling, burning sensations under electrodes (Poreisz et al., 2007; Boros et al., 2008; George and Aston-Jones, 2010) and other adverse effects including headache and pain during and after stimulation. All participants rated the unpleasantness of any scalp sensation using numeric analogue scales (NAS; e.g., 0 = no tingling to 10 = worst tingling imaginable).

### Data Management and Statistical Analysis

Peak-to-peak amplitude of 20 single-pulse MEPs were automatically calculated and averaged online for each time point of measurement, using a custom designed macro. Area under the curve of MEPs was also quantified off-line from the digitized averages of rectified EMG for conditioned and unconditioned stimuli in each trial by using a custom designed macro in Powerlab 8/30 software. The size of the conditioned MEP was expressed as a percentage of the unconditioned test MEPs in order to evaluate the effectiveness of ICI or ICF.

The differences in RMT recorded at the starting point of each experimental condition (T_pre_) were analyzed with one-way repeated measures analysis of variance (ANOVA) to detect any carry over effect. A two-way repeated measures ANOVA was performed to assess the effects of two independent variables (the peak-to-peak amplitude of MEPs, SICI, and IFC): experimental conditions with five levels, and measurement time with five levels on induced MEP amplitude. Mauchly’s test was used to assess the validity of the sphericity assumption for repeated measures ANOVA. Greenhouse-Geisser corrected significance values were used because sphericity could not be assumed (Meyers et al., 2006). In case of significant main effect, *post hoc* paired-sample two-tailed *t*-tests were performed using the least significant difference adjustment for multiple comparisons to evaluate the MEP, SICI, and ICF changes following the intervention at different time points of measurement and to compare baseline values with post-intervention measurements.

In order to assess whether participants were successfully blinded to the stimulation conditions (active or sham), Pearson’s chi-square was used. In addition, a one-way ANOVA was carried out on the mean values of rating scale recorded by questionnaire to assess any significant differences between the participants’ feelings during active and sham conditions. Statistical analyses were performed using SPSS software version 22. Means are reported ± standard error of measurement (SEM).

## Results

### Comparison of Baseline Values

One-way repeated measures ANOVA showed that there was no significant difference between baseline RMT at the starting point of all experimental conditions (*F*_4_ = 2.97, *p* = 0.09).

### The Effects of a-tDCS and a-tDCSUHCDS on M1 CSE

**Figure 3:**
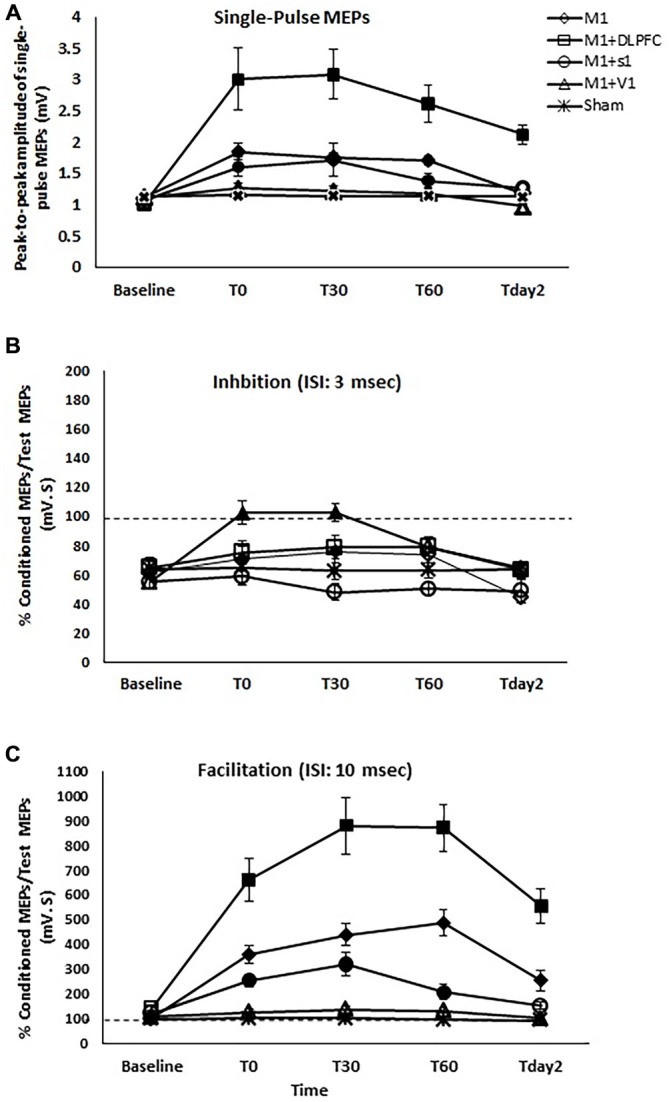
**The effects of different stimulation sites on the peak-to-peak amplitude of MEPs (A), SICI with ISI of 3 ms (B), and intracortical facilitation (ICF) (C) following a-tDCS of primary motor cortex (M1), a-tDCS_UHCDS_ of M1 and DLPFC, M1and primary sensory cortex (S1), M1and primary visual cortex (V1), and sham**. Filled symbols indicate significant deviation of the post-intervention MEP amplitude, SICI, and ICF compared to the baseline. Data are reported as mean ± SEM.

Two-way repeated measures ANOVA indicated significant main effects of experimental conditions (*F*_4_ = 18.41, *p* < 0.001), time (*F*_4_ = 33.55, *p* < 0.001), and the interaction of condition and time (*F*_16_ = 9.19, *p* < 0.001). MEP amplitude increased significantly following a-tDCS_UHCDS_ of M1-DLPFC compared to other experimental conditions in all time points of measurements (Figure 3A). As can be seen in Table 1, *t*-tests revealed a significant difference in MEP amplitude between stimulation of M1 and a-tDCS_UHCDS_ of M1–V1 condition at T_0_, T_30_, and T_60_, whilst no significant difference was found between sham and a-tDCS_UHCDS_ of M1–V1 condition. Similarly, no significant difference was detected between a-tDCS_UHCDS_ of M1–S1 and a-tDCS of M1 at one hour after intervention. The *post hoc* comparison also revealed a significant difference in MEP amplitude between a-tDCS_UHCDS_ of both M1–S1 and M1–DLPFC and other conditions at T_day2_ (Table 1). Comparing sham and four other experimental conditions revealed significant differences between all active tDCS conditions (except a-tDCS_UHCDS_ of M1–V1) and sham tDCS (Table 1).

**Table 1:** **Summary of post hoc comparisons of means differences at each time-point of measurement for the effects of conventional a-tDCS of M1 and unihemispheric concurrent dual-site a-tDCS on M1 corticospinal excitability dual a-tDCS stimulations on CSE of M1**.

		**1–2**	**1–3**	**1–4**	**1–5**	**2–3**	**2–4**	**2–5**	**3–4**	**3–5**	**4–5**
**Single-pulse TMS**	T_pre_	0.04	0.42	0.91	0.66	0.14	0.08	0.03	0.34	0.27	0.08
T_0_	0.004*	0.38	0.001*	0.03	0.000*	0.001*	0.000*	0.001*	0.02	0.34
T_30_	0.003*	0.91	0.000*	0.000*	0.016	0.001*	0.001*	0.005*	0.002*	0.49
T_60_	0.004*	0.06	0.001*	0.000*	0.001*	0.001*	0.000*	0.08	0.001*	0.82
T_day2_	0.000*	0.23	0.01	0.39	0.000*	0.000*	0.000*	0.004*	0.03	0.006

**SICI (ISI: 3 msec)**	T_pre_	0.96	0.40	0.23	0.58	0.45	0.33	0.005	0.98	0.28	0.122
T_0_	0.67	0.004*	0.26	0.31	0.047	0.168	0.001*	0.001*	0.006	0.32
T_30_	0.03	0.003*	0.02	0.004*	0.003*	0.03	0.17	0.000*	0.000*	0.005
T_60_	0.35	0.58	0.06	0.002*	0.96	0.003*	0.05	0.001*	0.08	0.07
T_day2_	0.001*	0.01	0.23	0.004*	0.001*	0.001*	0.000*	0.97	0.91	0.02
**ICF (ISI: 10 msec)**	T_pre_	0.04	0.09	0.12	0.14	0.01	0.04	0.07	0.09	0.17	0.32
T_0_	0.03	0.06	0.02	0.003*	0.001*	0.000*	0.000*	0.007	0.002*	0.05
T_30_	0.000*	0.001*	0.003*	0.000*	0.000*	0.000*	0.000*	0.07	0.004*	0.06
T_60_	0.000*	0.003*	0.003*	0.000*	0.000*	0.002*	0.000*	0.002*	0.004*	0.07
T_day2_	0.000*	0.07	0.12	0.034	0.001*	0.000*	0.000*	0.067	0.21	0.17

*The asterisks denote significant differences (*p* < 0.005)*. *1: Stimulation of M12: Stimulation of M1-DLPFC3: Stimulation of M1-S14: Stimulation of M1-V15: Sham tDCS*.

Comparing the MEP amplitudes baseline and post-intervention time points of measurement, the *post hoc* comparisons showed that there was significant differences between T_Pre_–T_0_ (*p* = 0.002), T_Pre_–T_30_ (*p* = 0.004), T_Pre_–T_60_ (*p* = 0.004) following a-tDCS_UHCDS_ of M1–S1, T_Pre_–T_0_ (*p* < 0.005), T_Pre_–T_30_ (*p* < 0.005), T_Pre_–T6_0_ (*p* < 0.005), and T_pre_−T_day2_ (*p* < 0.005) following a-tDCS_UHCDS_ of M1–DLPFC, and T_Pre_–T_0_ (*p* = 0.001), T_Pre_–T_30_ (*p* = 0.003), T_Pre_–T6_0_ (*p* = 0.003) following M1 a-tDCS. The results of *post hoc* comparisons are summarized in Figure 3A.

### The Effects of a-tDCS and a-tDCSUHCDS on SICI

Two-way repeated measures ANOVA showed significant effects of condition (*F*_4_ = 5.99, *p* = 0.001), Time (*F*_4_ = 21.24, *p* < 0.001), and interaction of time and condition (*F*_16_ = 6.55, *p* < 0.001) on SICI. *Post hoc* comparisons revealed no significant difference between a-tDCS_UHCDS_ of M1–DLPFC and a-tDCS of M1 at T_0_, T_30_, T_60_, and T_day2_ (Table 1). Significant differences in SICI were found between a-tDCS of M1 and a-tDCS_UHCDS_ of M1–S1 at T_0_ and T_30_ and between a-tDCS of M1 and sham tDCS at all post-intervention time points (Table 1). There was no significant SICI difference between a-tDCS_UHCDS_ of M1–V1 and sham condition in any time points of measurement.

*Post hoc* comparison also demonstrated that there was a significant difference between T_Pre_–T_0_ (*p* = 0.001) and T_Pre_–T_30_ (*p* = 0.001) following a-tDCS_UHCDS_ of M1–S1, and between T_Pre_–T_0_ (*p* = 0.004) and T_Pre_–T_30_ (*p* = 0.004) following M1 a-tDCS. No significant SICI alteration was found at any time-points following a-tDCS_UHCDS_ of M1–DLPFC, M1–V1, or sham condition (Figure 3B).

### The Effects of a-tDCS and a-tDCSUHCDS on ICF

Two-way repeated measures ANOVA found significant main effects of condition (*F*_4_ = 36.74, *p* < 0.001), time (*F*_4_ = 65.31, *p* < 0.001), and interaction of condition × time (*F*_16_ = 21.29, *p* < 0.001) on ICF. *Post hoc* comparisons revealed significant ICF differences between a-tDCS_UHCDS_ of M1–DLPFC and all other conditions at all time points of measurement (Table 1). Significant differences in ICF were also found between a-tDCS_UHCDS_ of M1–S1 and a-tDCS of M1 at T_30_ and T_60_ (Table 1). There was a significant difference in ICF between sham and other active conditions except a-tDCS_UHCDS_ of M1–V1 (Table 1).

Comparing post-intervention and baseline values, the result showed significant difference between T_Pre_–T_0_ (*p* = 0.001), T_Pre_–T_30_ (*p* = 0.001), T_Pre_–T6_0_ (*p* < 0.005), and T_pre_–T_day2_ (*p* = 0.001) following a-tDCS_UHCDS_ of M1–S1. Significant differences were also found between T_Pre_–T_0_ (*p* < 0.005), T_Pre_–T_30_ (*p* < 0.005), T_Pre_–T6_0_ (*p* < 0.005), and T_pre_–T_day2_ (*p* < 0.005) following a-tDCS_UHCDS_ of M1–DLPFC, and T_Pre_–T_0_ (*p* = 0.004), T_Pre_–T_30_ (*p* = 0.003), T_Pre_–T6_0_ (*p* = 0.004) following M1 a-tDCS. No significant difference in ICF was found following a-tDCS_UHCDS_ of M1–V1 or sham condition at any time point (Figure 3C).

### Safety and Side Effects of a-tDCSUHCDS

Participants’ experiences were recorded at the beginning, during and at the end stage of the intervention. The averaged sensation score recorded during the intervention is summarized in Table 2. The only reported sensations related to the anode were itching and tingling. Based on the result, the most sever tingling (4.3 ± 0.2) and itching (3.1 ± 0.64) were recorded under the anode electrode at the beginning of M1–S1 condition. Itching and tingling under the cathode electrode were also the most commonly reported side effects. One of the participants reported a burning sensation at the beginning of a-tDCS_UHCDS_ of M1–S1. No adverse effect related to a-tDCS_UHCDS_ or a-tDCS was detected during the follow-up measurements.

**Table 2:** **Participant’s sensation scores during experimental conditions**.

		Anode electrode					Reference electrode				
		M1	M1-DLPFC	M1-S1	M1-V1	Sham	M1	M1-DLPFC	M1-S1	M1-V1	Sham
Tingling	Beginning	3.6 ± 0.21	3.9 ± 0.34	4.3 ± 0.2	2.9 ± 0.27	2.1 ± 0.16	1.5 ± 0.13	1.8 ± 0.12	2.1 ± 0.13	1.7 ± 0.22	1.4 ± 0.19
	Middle	2.1 ± 0.18	2.8 ± 0.15	1.4 ± 0.31	2.1 ± 0.14	1.4 ± 0.10	1.1 ± 0.18	0.7 ± 0. 10	1.4 ± 0.10	1.2 ± 0.16	1.0 ± 0.07
	End	–	1.1 ± 0.19	1.1 ± 0.45	1.7 ± 0.21	0.8 ± 0.10	0.5 ± 0.27	0.6 ± 0.11	0.8 ± 0.24	0.9 ± 0.19	0.5 ± 0.1
Itching	Beginning	2.9 ± 0.09	3.0 ± 0.36	3.1 ± 0.64	1.3 ± 0.29	1.2 ± 0.21	1.1 ± 0.12	1.1 ± 0.09	1.2 ± 0.15	1.8 ± 0.11	1.1 ± 0.08
	Middle	1.3 ± 0.28	1.9 ± 0.03	2.6 ± 0.12	0.9 ± 0.15	0.8 ± 0.14	0.4 ± 0.16	0.6 ± 0.25	0.6 ± 0.17	1.2 ± 0.15	0.8 ± 0.12
	End	–	0.7 ± 0.1	1.2 ± 0.52	0.7 ± 0.23	–	0.1 ± 0.20	–	0.3 ± 0.06	0.9 ± 0.12	0.6 ± 0.09
Burning	Beginning	–	–	0.45 ± 0.1	–	0.23 ± 0.07		–	–	–	–
	Middle	–	–	0.31 ± 0.07	–	0.2 ± 0.03		–	–	–	–
	End	–	–	–	–			–	–	–	–
Not tolerated	Beginning	–	–	–	–			–	–	–	–
	Middle	–	–	–	–			–	–	–	–
	End	–	–	–	–			–	–	–	–

*The values are rated using the NAS. 0 is rated as no sensation and 10 rated as the worst sensation imaginable. The sensations are recorded during three phases of stimulation: Beginning (0–7 min of stimulation), Middle (7–14 min of stimulation), End (14–20 min of stimulation). Sensations under both active (anode) and reference (cathode) electrodes were recorded during a-tDCS of M1, S1, DLPFC and sham a-tDCS. Scores are reported*.

The participant’s judgment on the stimulation conditions is summarized in Table 3. Pearson’s chi square showed no significant differences between the active and sham conditions (χ^*2*^(4, *n* = 12) = 6.75, *p* = 0.15), demonstrating that participants were not able to determine the type of stimulation. The majority of participants were properly blinded and the active or sham conditions were correctly guessed just in 16% of conditions (excluding the “Cannot say” responses).

**Table 3:** **The judgements of participants on the stimulation condition**.

		Actual testing conditions (*n* = 12)					
		a-tDCS of M1	a-tDCS_UHCDS_ of M1-DLPFC	a-tDCS_UHCDS_ of M1–S1	a-tDCS_UHCDS_ of M1–V1	Sham	Total
Perceived stimulation	Active	2	2	3	1	4	12
	Sham	4	4	4	4	2	18
	Cannot say	6	6	5	7	6	30
	Total	12	12	12	12	12	60

The results of one-way ANOVA indicated that sensations were significantly different across the conditions (*F*_(4,47)_ = 7.36, *p* = 0.01). The *post hoc* comparisons showed that there was no significant difference between sensation of participants in sham and active conditions under the cathode electrode (except between sham and active a-tDCS_UHCDS_ of M1–S1 stimulation (*p* = 0.004) at the End stage of stimulation). Under the reference electrode, there was no significant difference between active and sham conditions.”

## Discussion

### Comparison of Baseline Values

All baseline RMT values remained unchanged at the starting point of all experimental conditions, meaning the washout period was adequate and any possibility of carry over effect from previous interventions on the same participants is refuted.

### The Effects of a-tDCSUHCDS on M1 CSE

Our study was designed to assess the effects of concurrent stimulation of ipsilateral M1 and DLPFC on M1 CSE. Compared to a-tDCS of M1, we found that a-tDCS_UHCDS_ of M1–DLPFC induces larger M1 CSE (~1.5 times) which lasted at least 24 h. We also found that a-tDCS_UHCDS_ of M1–S1 increased CSE of M1 for 30 min, whilst the effects of a-tDCS on M1 lasted for one hour; there was no significant change in the size of MEPs in these two conditions. We hypothesized that concurrent stimulation of M1 and the other sites of the same hemisphere considerably increases M1 CSE. Our findings support this hypothesis in part. The results are in line with those of previous studies, which reported that M1 a-tDCS increased M1 CSE for one hour (Nitsche and Paulus, 2000, 2001; Bastani and Jaberzadeh, 2013). Compared to M1 stimulation, a-tDCS_UHCDS_ of M1-DLPFC increased the size of MEPs for at least for 24 h. This study is the first to assess the effects of unihemispheric concurrent dual site stimulation of target areas of the brain, so further research is needed to support or disprove our results. However, considerable larger MEPs following a-tDCS_UHCDS_ of M1-DLPFC, lasting for at least 24 h, is an extremely valuable clinical finding and should be explored further in future studies.

Comparison of the results from the conventional M1 a-tDCS and a-tDCS_UHCDS_ of M1–DLPFC and other functionally connected pairs indicated that concurrent stimulation of M1-DLPFC is a more effective technique to increase M1 CSE. The efficacy of a-tDCS_UHCDS_ of M1–DLPFC on CSE enhancement is more likely to be site specific, which is caused due to the effect of concurrent stimulation of functionally connected sites of M1. With some reasons, the findings in this study rule out the doubling of total charge as a driven source for the observed changes. First, a-tDCS_UHCDS_ of M1–V1 had no effect on M1 CSE. Second, there was no significant difference between active and sham a-tDCS_UHCDS_. Third, a-tDCS_UHCDS_ of M1–S1 had similar effects on the size of MEPs to those of standard a-tDCS but with reduced durability. In a recent study conducted by our group, we applied a-tDCS over M1, S1, and DLPFC separately and found that CSE of M1 was significantly increased by a-tDCS of M1 or DLPFC (Vaseghi et al., 2015b). Moreover, some anatomical studies indicate that the premotor cortex is divided into dorsal and ventral parts and the dorsal part sends its output to the M1 and spinal cord and receives prominent input from DLPFC (Dum and Strick, 1991; He et al., 1993). The attention modulation signals from the DLPFC and motor preparation information from the dorsal part of the premotor cortex are received by the M1 (Bunge et al., 2001; Nitsche and Paulus, 2001; Van Ryckeghem et al., 2013). As a result, compared to stimulation of M1, a-tDCS_UHCDS_ of M1–DLPFC may activate the DLPFC-premotor-primary motor pathway (Hoshi, 2006; Bracht et al., 2012) and increase M1 excitability. In contrast, inhibitory and fast-spiking interneurons named Vasointestinal Peptides (VIPs) in the superficial layers of S1 project to M1 pyramidal neurons; they account for the most GABAergic interneurons in S1 and target the distal dendrites of pyramidal cells in M1 (Lee et al., 2010, 2013; Rudy et al., 2011). It is possible that concurrent stimulation of M1 and S1 with a-tDCS_UHCDS_ might activate VIP interneurons that in turn increase the size of MEPs and promote their long-lasting effects. The effect of excitability changes in V1 on CSE of M1 has not been investigated; however, Pirulli et al. (2014) found that V1 excitability changes have opposite effects on motor performance. They applied cathodal tDCS on V1, which led to motor performance improvement, and concluded that possible inhibitory compensatory circuits in V1 are inhibited by c-tDCS, resulting in motor performance improvement (O’Shea et al., 2007; Jacobson et al., 2012). Consequently, it is possible that in our experiments stimulation of V1 with M1 increased the inhibitory effects of those inhibitory circuits, which led to suppression of the effects of stimulation of M1, and subsequently a-tDCS_UHCDS_ of M1–V1 had no effects on CSE of M1.

### The Effects of a-tDCSUHCDS on SICI

In our study, SICI reduced for 60 min after a-tDCS of M1, which supports our hypothesis and is consistent with some previous studies of the effects of a-tDCS of M1 (Liepert et al., 1998; Hummel et al., 2005; Nitsche et al., 2005; Kidgell et al., 2013b). Few researchers have described the effects of a-tDCS on the GABAergic inhibitory system (Nitsche et al., 2005; Hummel et al., 2010) but many researchers are studying different approaches to find the most efficient method with a reasonably long-lasting effect. Nitsche et al. (2005) found a significant increase in SICI lasted for at least 30 min following 13 min of 1 mA a-tDCS (Nitsche et al., 2005). In contrast, another recent study suggested that SICI reduces for 30 min following a-tDCS of M1 (Kidgell et al., 2013a). The authors applied a-tDCS over M1 with a range of current intensities, and concluded that a-tDCS of M1 reduces SICI independently of current intensity. Yet again, Batsikadze et al. (2013) observed no significant changes in SICI following 20 min of 2 mA a-tDCS.

We demonstrated that SICI was reduced for at least 30 min after a-tDCS_UHCDS_ of M1–S1 and there was significant difference between this condition and a-tDCS of M1, supporting our hypothesis. These finding may suggest that both conventional a-tDCS and a-tDCS_UHCDS_ of M1–S1 can increase the excitability of intracortical inhibitory interneurons and as a result, reduce SICI. It can be concluded that CSE enhancement is independent of stimulation site in the dominant hemisphere. In addition, significant differences between sham and conventional a-tDCS of M1, a-tDCS_UHCDS_ of M1–S1 indicate that the results observed are due to the real effects of conventional a-tDCS and a-tDCS_UHCDS_.

We also found that a-tDCS_UHCDS_ of M1–V1 induced no significant changes in SICI and there was no significant difference between sham and a-tDCS_UHCDS_ of M1–V1. It is suggested that increasing the current intensity in the same hemisphere is not the main reason behind the CSE enhancement of M1; functional connectivities probably play an important role in this regard.

This study is the first to investigate the effect of concurrent stimulation of M1 and another site in the same hemisphere on the M1 CSE. It seems that conventional stimulation of M1 and a-tDCS_UHCDS_ of M1–DLPFC and M1–S1 reduce GABAergic intracortical inhibition, which can be interpreted as disinhibition of corticospinal neurons, resulting in increased CSE.

### The Effects of a-tDCSUHCDS on ICF

We showed that a-tDCS of M1 increased the level of ICF in the stimulated area. In addition, comparing single- and double-site conditions showed that a-tDCS_UHCDS_ of M1–DLPFC increased the level of ICF to triple that of a-tDCS of M1, and this effect lasted for 24 h after the intervention. This finding supports our hypothesis. Moreover, significant differences between active and sham conditions demonstrated that the results are not due to the placebo effect.

Some evidence supports an increase of ICF after a-tDCS of M1 (Chen, 2004; Nitsche et al., 2005; Batsikadze et al., 2013). In line with our results, it has been shown that ICF increases immediately after a-tDCS lasted for 90 min (Batsikadze et al., 2013). In contrast, Ogata et al. (2007) reported that a-tDCS of M1 has no significant influence on ICF or SICI (Ogata et al., 2007). Differences between Ogata et al.’s methods of conditioning and test stimulus intensity and our own probably explain these results.

Given that glutamate and NMDA receptors are involved in mediating ICF (Ziemann et al., 1996, 1998; Chen et al., 1998), it can be concluded that glutamergic and NMDA receptor concentration in the M1 intensifies following a-tDCS of M1. Since the present study is the first to investigate a-tDCS_UHCDS_ effects on CSE, the results cannot be compared to other studies directly. However, regarding the role of DLPFC in motor functions (Bedwell et al., 2014; Van Snellenberg et al., 2014; Harding et al., 2015), it can be suggested that a-tDCS_UHCDS_ M1–DLPFC might stabilize the tDCS-induced NMDA-receptor-dependent excitability enhancement in M1, resulting in raised ICF.

As can be seen in Figure 3, the level of ICF decreased following a-tDCS_UHCDS_ of M1–S1 compared to a-tDCS of M1. Therefore, our hypothesis is not supported. No researchers have investigated the effects of concurrent stimulation of M1 and S1 in the same hemisphere, but in a recent study, M1 CSE enhancement was found with 30 min delay following a-tDCS of S1 (Vaseghi et al., 2015b). These authors concluded that the inhibitory effects of VIP interneurons on M1 probably increased after a-tDCS of S1 (Vaseghi et al., 2015b). Thus, one possible explanation for our own results is that increasing the activity of inhibitory VIP interneurons in S1 has an effect on CSE of M1, which controls the excitability enhancement of M1 following a-tDCS_UHCDS_ of M1–S1.

### Safety and Side Effects of a-tDCSUHCDS

Based on the results, participants were successfully blinded to the experimental conditions. They were not able to distinguish the active or sham conditions (except in ending stage of active M1-S1 a-tDCS_UHCDS_ condition). No significant difference in rating scales was also found under the reference electrodes in sham and active conditions. In addition, minimal side effects following a-tDCS_UHCDS_ suggest that stimulation of two functional areas of the same hemisphere with two separated tDCS devices is a safe approach in healthy individuals. The participants’ tolerance for a-tDCS_UHCDS_ with small electrodes was comparable with that for the conventional approach with larger electrodes. Similar to previous studies (Gandiga et al., 2006; Brunoni et al., 2011), general discomfort (itching/tingling) was the most frequently recorded side effect and just one participant of 12 reported a slight burning feeling. In addition, Poreisz et al. (2007) investigated the tDCS side effects over a large number of participants in both healthy and patient groups, while M1, S1, DLPFC, and visual cortex were stimulated. The results demonstrated that mild tingling and tingling were the most common sensations in healthy adults and there was no significant difference between participants’ sensation after stimulation of different cortical targets (Poreisz et al., 2007).

#### Limitations of the Study

Our study has some limitations. First, the duration of the CSE stimulation effect of a-tDCS_UHCDS_ was only assessed up to 24 h after intervention. Longer follow-up is required to properly evaluate the lasting effect of a-tDCS_UHCDS_ of M1–DLPFC, and such data will be valuable for future studies investigating an optimal approach to enhance CSE of M1. Second, the effects were evaluated in young participants (less than 35 years); older individuals may respond differently to a-tDCS_UHCDS_. Third, we utilized a conventional electrode montage with active electrodes (anode) over target stimulation areas and reference electrodes (cathode) over the contralateral supraorbital area (subgenual cortex). Regarding the functional connectivity between the subgenual cortex and the stimulated sites in this study, it is possible that the position of reference electrodes affect the level of CSE.

### Suggestions for Future Research

Our results and the known functional connectivities between M1 and other cortical areas of the brain involved in motor learning, including the posterior parietal cortex, premotor cortex and supplementary motor area, suggest that the effect of a-tDCS_UHCDS_ of these areas on M1 CSE should be investigated. In addition, more studies are required to fully characterize the effects of a-tDCS_UHCDS_ on CSE of M1. For instance, the effects of a-tDCS_UHCDS_ application time, current intensity, and electrode size should be systematically studied to improve our understanding of these phenomena and their interactions. Furthermore, additional pharmacological experiments using receptor agonists/antagonists are needed to determine the exact mechanism behind the efficacy of a-tDCS_UHCDS_. It is also recommended that the effects of cathodal tDCS_UHCDS_ on CSE of M1 be investigated. Such data will clarify the connectivities of the cortical areas of the brain.

## Conclusion

We found that a-tDCS_UHCDS_ of M1-DLPFC not only considerably enhances M1 CSE (three fold) compared to the conventional a-tDCS approach, but extends the effects for at least 24 h. Further development of this new approach is likely to produce an efficient therapeutic neurorehabilitation strategy for pain treatment in patients with chronic pain or for motor performance improvement in stroke or multiple sclerosis patients.

## Author Contributions

The Corresponding author of this manuscript is “BV”, and “SJ” and “MZ” contribute in preparation of the current manuscript. The current study is a part of PhD thesis of the corresponding author. So, “SJ”, as the main supervisor, and “MZ”, as the co-supervisor, helped the corresponding author to design the study, interpret the results of study, and provide feedback on final conclusion and final draft of the manuscript.

With the submission of this manuscript, I would like to undertake that:

- All authors of this research paper have directly participated in the planning, execution, or analysis of this study;
- All authors of this paper have read and approved the final version submitted;
- The contents of this manuscript have not been copyrighted or published previously;
- The contents of this manuscript are not now under consideration for publication elsewhere;
- The contents of this manuscript will not be copyrighted, submitted, or published elsewhere, while acceptance by the Journal is under consideration;
- There are no directly related manuscripts or abstracts, published or unpublished, by any authors of this paper;
- My Institute’s (Monash University) representative is fully aware of this submission.

## Conflict of Interest Statement

The authors declare that the research was conducted in the absence of any commercial or financial relationships that could be construed as a potential conflict of interest.

## Footnotes

^1^http://www.anzctr.org.au/
